# Everybody's Column

**Published:** 1892-02-20

**Authors:** 


### EVERYBODY'S COLUMN.

ONE of the latest additions to the Zoological Gardens is a Japanese curiosity in the shape of some white mice, which are peculiar for their feline habit of running after their own tails!

---

THERE is a prevailing belief, and rightly so, that the mumps are decidedly catching. It has been left to Judge Shand to throw fresh light on the infectious nature of this unpleasant and disfiguring complaint, his experience of which seems to have been peculiarly unfortunate. In giving judgment recently at Liverpool in a case in which the complaint formed the *casus belli*, his Honour announced feelingly, "One can catch it (the mumps) almost in five minutes by looking at a person, as I know by my own experience."

ONE part each of lactic acid and salicylic acid, mixed with eight parts of collodion is recommended as an effective application either to corns or warts, causing their removal very speedily. The discoverer of this simple remedy, if it accomplishes all that is asserted on its behalf, deserves a testimonial.

---

How delighted elderly ladies will be to hear of a simple method of restoring their long-lost youth. If the *Medical Press* is not so ungallant as to be having a laugh at their expense, this much-desired end can be attained by rubbing lanolin well into the skin. The result of this treatment is said to be the smoothing out of all those tell-tale folds which are the despair of those upon whom time is setting his mark.

